# P-1474. Development of an HIV vaccine based on 2C6, an antibody that targets a conformational gp41 epitope with cross-clade recognition and robust antibody dependent cell cytotoxicity

**DOI:** 10.1093/ofid/ofaf695.1660

**Published:** 2026-01-11

**Authors:** Marvin Petion, Jonathan Lovell, Yiting Song, Shiqi Zhou, Mark D Hicar

**Affiliations:** University at Buffalo, Buffalo, New York; University at Buffalo, Buffalo, New York; University at Buffalo, Buffalo, New York; SUNY-Buffalo, Buffalo, New York; University at Buffalo, Buffalo, New York

## Abstract

**Background:**

Since the Human Immunodeficiency Virus (HIV) pandemic began, roughly 80 million people have been infected, resulting in over 40 million deaths. HIV is a blood-borne disease that causes immunodeficiency by targeting CD4 T-cells. To date, there has not been a successful vaccine against HIV. In our prior studies, we have described novel epitopes on gp41 that are potential targets of future vaccine development. The 2C6 antibody (Ab) binds to a conformational epitope pocket between gp41 protomers and recognizes trimeric HIV envelope including SOSIP constructs. 2C6 has robust cross-clade Ab-dependent cell cytotoxicity (ADCC) activity and readily recognizes HIV clades A-E. Initial studies suggest that a 25-mer peptide represents enough of the structure of the 2C6 epitope to be recognized. In this study, we sought to assess if 2C6 would be a viable vaccine candidate by performing serum competition and initial murine vaccination.

**Figure:**
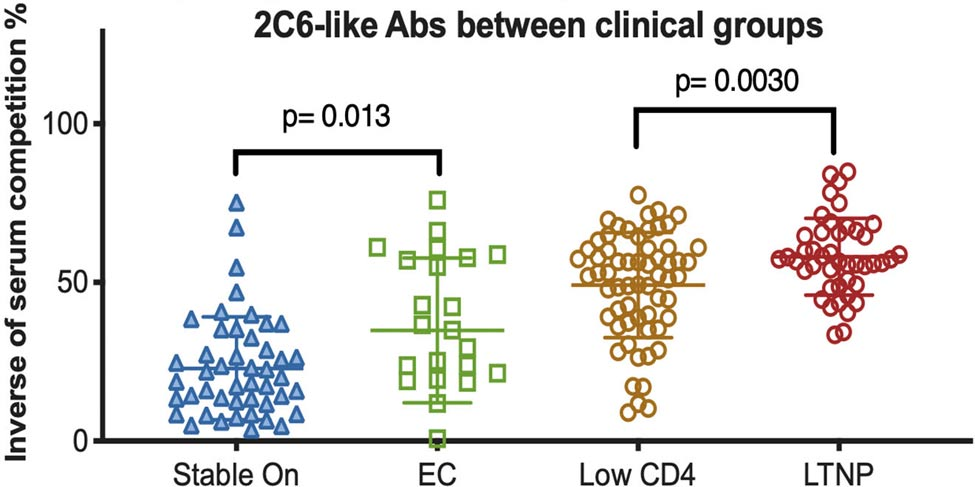
2C6 antibody competition with HIV + patient serum 2C6 in LTNP and Elite Controllers (ECs). Comparison between groups with suppressed viral loads (Stable On therapy/EC) and those with appreciable viral loads (Low CD4s and LTNPs) show 2C6 correlates with control.

**Figure:**
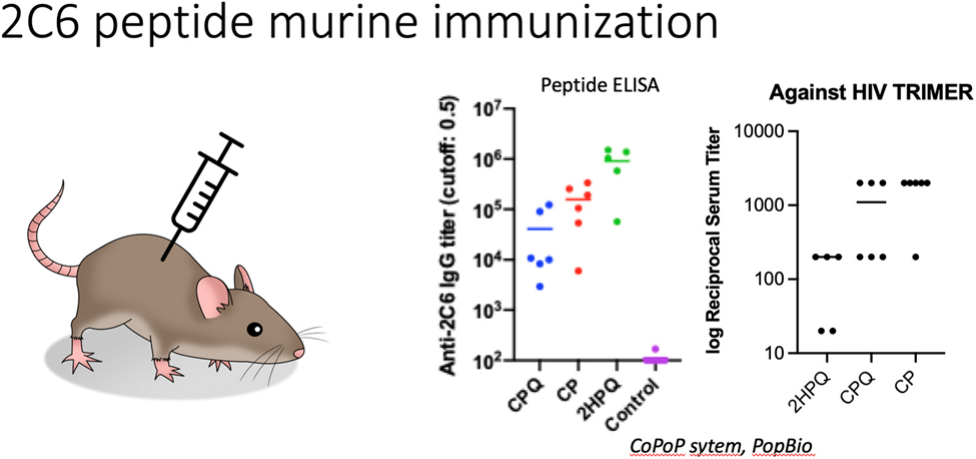
Murine Vaccination shows recognition of peptide and SOSIP trimer Initial murine studies (4-6) mice were vaccinated with CoPoP formulation (lipid particle- CP), squalene adjuvants (Q), or without cobalt particle incorporation (2HP).

**Methods:**

Serum samples were obtained from HIV+ clinical groups: Elite controllers (EC), long-term non-progressors (LTNPS), stable CD4 counts on therapy, and those off therapy. Preliminary murine studies were performed using a His-tagged peptide that included the 25-amino acid peptide that formed the base of 2C6 epitope. Mice were immunized with various formulations including Cobalt-Porphyrin-Phospholipid (CoPoP or CP) formulation and a saponin based adjuvant.

**Results:**

In Ab/serum competition assays, Abs targeting the 2C6 epitope were enriched in LTNPs and in ECs in comparison to their control groups implying an effect of on viral control by Abs targeting the 2C6 epitope. Recognition of SOSIP trimers were particularly improved by CoPoP display of the 25-mer peptide.

**Conclusion:**

The results show that our vaccine can induce an immune response that replicates trimer binding, with Porphyrin-Phospholipids and Saponin-derived adjuvants being the best formulations. Ongoing studies are addressing a panel of antigens to assess improved recognition and assessing if this vaccine can raise Abs that have ADCC.

**Disclosures:**

Jonathan Lovell, PhD, POP Biotechnologies: Ownership Interest Mark D. Hicar, MD/PhD, Pfizer: site-PI for vaccine study on Lyme

